# Role of ascorbic acid in the regulation of epigenetic processes induced by *Porphyromonas gingivalis* in endothelial-committed oral stem cells

**DOI:** 10.1007/s00418-021-02014-8

**Published:** 2021-08-02

**Authors:** Jacopo Pizzicannella, Guya Diletta Marconi, Simone Guarnieri, Luigia Fonticoli, Ylenia Della Rocca, Fani Konstantinidou, Thangavelu Soundara Rajan, Valentina Gatta, Oriana Trubiani, Francesca Diomede

**Affiliations:** 1 “Ss. Annunziata” Hospital, ASL 02 Lanciano-Vasto-Chieti, Via dei Vestini, 29, Chieti, 66100 Italy; 2 Department of Medical, Oral and Biotechnological Sciences, University “G. D’Annunzio” Chieti-Pescara, Via dei Vestini, 31, Chieti, 66100 Italy; 3 Department of Neuroscience, Imaging and Clinical Sciences, University “G. D’Annunzio” Chieti-Pescara, Via dei Vestini, 31, Chieti, 66100 Italy; 4 Center for Advanced Studies and Technology (CAST), University “G. D’Annunzio” Chieti-Pescara, Via Luigi Polacchi,19, Chieti, 66100 Italy; 5 Department of Innovative Technologies in Medicine & Dentistry, University “G. D’Annunzio” Chieti-Pescara, Via dei Vestini, 31, Chieti, 66100 Italy; 6 Department of Psychological, Health and Territorial Sciences, University “G. D’Annunzio” Chieti-Pescara, Via dei Vestini, 31, Chieti, 66100 Italy; 7 Department of Biotechnology, Karpagam Academy of Higher Education, Pollachi Main Road, Eachanari Post, Coimbatore, 641021 Tamil Nadu India

**Keywords:** Periodontitis, Epigenetic modifications, Lipopolysaccharides, Human gingival mesenchymal stem cells, Endothelial differentiation

## Abstract

Periodontitis is a common inflammatory disease that affects the teeth-supporting tissue and causes bone and tooth loss. Moreover, in a worldwide population, periodontal disease is often associated with cardiovascular diseases. Emerging studies have reported that one of the major pathogens related to periodontitis is *Porphyromonas gingivalis* (*P. gingivalis*), which triggers the inflammatory intracellular cascade. Here, we hypothesized a possible protective effect of ascorbic acid (AA) in the restoration of the physiological molecular pathway after exposure to lipopolysaccharide derived from *P. gingivalis* (LPS-G). In particular, human gingiva-derived mesenchymal stem cells (hGMSCs) and endothelial-differentiated hGMSCs (e-hGMSCs) exposed to LPS-G showed upregulation of p300 and downregulation of DNA methyltransferase 1 (DNMT1), proteins associated with DNA methylation and histone acetylation. The co-treatment of AA and LPS-G showed a physiological expression of p300 and DNMT1 in hGMSCs and e-hGMSCs. Moreover, the inflammatory process triggered by LPS-G was demonstrated by evaluation of reactive oxygen species (ROS) and their intracellular localization. AA exposure re-established the physiological ROS levels. Despite the limitations of in vitro study, these findings collectively expand our knowledge regarding the molecular pathways involved in periodontal disease, and suggest the involvement of epigenetic modifications in the development of periodontitis.

**Supplementary Information:**

The online version contains supplementary material available at 10.1007/s00418-021-02014-8.

## Introduction

Periodontal disease is widespread, potentially affecting up to 90% of the world’s population. Periodontal diseases, including gingivitis and periodontitis, possess an essential bacterial multifactorial etiology, with the interaction of genetic and environmental factors (Pihlstrom et al. 2005).

Periodontitis is induced by microbial biofilm and caused by host-mediated inflammation, which leads to collateral tissue damage and clinical attachment loss (Hajishengallis et al. 2012). The oral microbiota is composed of more than 700 different species of microorganisms (Aas et al. 2005). Socransky defined the “red complex,” which includes *Porphyromonas gingivalis*, *Treponema denticola*, and *Tannerella forsythia*, as the major pathogens responsible for periodontitis progression (Socransky et al. 1998).

In particular, *P. gingivalis* can interfere with host immune responses and alter the symbiosis between the local oral bacteria, and can also play a key role in pathogen colonization and periodontitis pathogenesis (Hajishengallis 2014).

Lipopolysaccharide (LPS) is a major component of the cell wall of Gram-negative bacteria, including the periodontal pathogen *P. gingivalis*. LPS is well known for its toxicity and ability to facilitate the host inflammatory response by stimulating Toll-like receptor 4 (TLR4) (Lien et al. 2000), playing a strong pathogenic role in periodontal tissues that leads to a favorable environment for pathogens responsible for sustaining and ultimately causing periodontal disease progression (Papapanou and Susin 2017). Furthermore, common types of periodontal disease and its related infections have been associated with cardiovascular disease (CVD), adverse pregnancy outcomes, stroke, lung disease, and diabetes (Pihlstrom et al. 2005). Uncontrolled periodontal infections can potentially affect vascular endothelial cells with the upregulation of systemic levels of inflammation, and thus contribute to endothelial dysfunction and the onset of atherosclerosis and CVD (Vapaatalo and Mervaala 2001; Aarabi et al. 2018; Priyamvara et al. 2020).

*P. gingivalis* is gaining recognition as a contributor to CVD such as aortic aneurysm and atherosclerosis (Nakano et al. 2011; Kebschull et al. 2010).

A large number of studies have investigated the mesenchymal stem cell (MSC) response to inflammatory events triggered by different stimuli and treated with antioxidant agents. In recent years, oral tissues such as dental pulp, dental follicle, apical papilla, periodontal ligament, gingiva, and palatine tonsil have been identified as alternative sources for adult MSCs (Andrukhov et al. 2019; Rajan et al. 2017; Bianchi et al. 2017).

Among these, MSCs derived from the human gingiva (hGMSCs) have shown the ability to differentiate into mesengenic lineages and to protect against infectious diseases due to their immunomodulatory properties (Li et al. 2014; Hajishengallis 2015; Rajan et al. 2017; Marconi et al. 2020). It must be mentioned here that hMSCs play an active role in the immune response by interacting with natural killer cells, dendritic cells, B lymphocytes and T lymphocytes. In this way, hMSCs prevent improper activation of T cells and stem the immune response during healing (Papapanou and Susin 2017; Libro et al. 2016a, b; Pisciotta et al. 2020).

Ascorbic acid (AA) is involved in the reduction of reactive oxygen species (ROS) and performs an anti-senescence role (Ballerini et al. 2017). AA, added to cell culture, had been shown to increase cell proliferation and cell pluripotency markers (Frei et al. 1989; Fujisawa et al. 2018), and to promote MSC regeneration of periodontal ligament tissue, reducing inflammation (Wei et al. 2012).

Periodontal disease progression is regulated by bacteria and their products, which may lead to DNA methylation (Diomede et al. 2017; Moore et al. 2013). Epigenetics can be defined as the study of genetic regulatory mechanisms which concern phenotype changes without involving alterations in an individual's DNA sequence. Epigenetic causally related modifications can be responsible for expressing or silencing genes and, as a consequence, determine protein production. These alterations include events such as DNA methylation, histone modification, and microRNA regulation. DNA methylation is a process in which additional methyl groups are added to the DNA, and occurs in regions mostly known as CpG islands, in which a cytosine nucleotide is located next to a guanine nucleotide, linked by a phosphate. Inserting methyl groups changes the structure of DNA, modifying a gene's interactions with the transcription-associated machinery within a cell’s nucleus (Egger et al. 2004). Furthermore, the effects of DNA methylation are often observed in terms of transcriptional modulation; hypermethylation is known to result in a decrease in gene expression, while hypomethylation is usually related to upregulation of transcription (Campos et al. 2016).

Among common oral inflammatory conditions, the presence of epigenetic modifications in proinflammatory cytokines such as Fas-associated death domain protein (FADD), interleukin-12 subunit beta (IL-12B), and interleukin-4 receptor (IL-4R) has been associated with periodontal disease (Schulz et al. 2016). In addition, studies have reported the involvement of DNA methylation events in apical periodontitis-related genes, suggesting epigenetic regulation as a potential regulatory mechanism in the inflammatory response (Mehrazarin et al. 2017). Moreover, considering that AA is an important epigenetic modulator (Lee Chong et al. 2019), we also studied its impact on the promoter methylation status of *CCL5* and *ICAM1* genes as a potential regulatory mechanism in the inflammatory response. Both genes are known to be related to inflammatory response also associated with oral pathological conditions (Wichnieski et al. 2019).

The present study used endothelial-committed oral stem cells as a model to investigate the relationship between periodontal disease and CVD by exposing the cells to lipopolysaccharide derived from *P. gingivalis* (LPS-G).

The aim of the present work was to investigate the anti-inflammatory and epigenetic modulatory properties of AA in LPS-G-exposed hGMSCs and e-hGMSCs. Expression of epigenetic markers p300 and DNA methyltransferase 1 (DNMT1), and ROS level were studied.

## Materials and methods

### Ethical statement

The study protocol was approved by the Ethics Committee at the “G. d’Annunzio,” Chieti, Italy (n. 266/17). The Department of Medical, Oral and Biotechnological Sciences and the Laboratory of Stem Cells and Regenerative Medicine are certified according to the quality standard ISO 9001:2008 (certificate no. 32031/15/S). All patients who were recruited for this study completed and signed an informed consent form. Except following the ethical standards of the institutional and/or national research committee, all procedures act in accordance with the 1964 Declaration of Helsinki and its subsequent reviews.

### hGMSC culture establishment

Patients recruited for this study had to undergo surgical procedures to initiate orthodontic treatment. Gingival tissue was obtained from biopsy during oral surgery procedures. All recruited patients were in good health and did not suffer from oral cavity diseases. After tissue collection, the gingival tissue fragments were crumbled and washed five times with phosphate-buffered saline (PBS) solution (Lonza, Basel, Switzerland). Following these washes, the fragments were placed in a culture dish inside an incubator at 37 °C with a humidified atmosphere at 5% CO_2_ in air. Chemically defined mesenchymal stem cell growth medium (MSCGM-CD, Lonza) was used as a culture medium and was changed every 2 days to stimulate the growth of human GMSCs. After 2 weeks of culture, isolated cells were migrated spontaneously from the explants. To evaluate the cell morphology and the capacity to adhere on a plastic substrate, hGMSCs at second passage were seeded on petri dishes and stained with toluidine blue solution, as reported previously (Mazzatenta et al. 2014).

### hGMSC characterization by FACS analysis

After washing in PBS, hGMSCs were analyzed for the expression of CD14, CD34, CD45, CD73, CD90, and CD105. Briefly, cells positive for CD45, CD73, and CD90 were detected with anti-human antibodies conjugated with fluorescein isothiocyanate, while those expressing CD14, CD34 and CD105 were detected with human antibodies conjugated with phycoerythrin. Subsequently, the Fluorescence Activated Cell Sorter (FACS) Star Plus flow cytometry system with CellQuest software (Becton Dickinson, Mountain View, CA, USA) was used for the analysis. All reagents used for flow cytometry were purchased from Becton Dickinson.

### Analysis of hGMSC mesenchymal differentiation (colorimetric detection and RT-PCR)

Since hGMSCs are able to differentiate into mesengenic lines, such as adipogenic and osteogenic commitment, colorimetric detection and reverse transcription polymerase chain reaction (RT-PCR) were used to confirm this differentiation property. Specifically, to induce the adipogenic commitment, the hGMSCs were cultured in a 24-well plate with a density of 2 × 10^4^ cells/well with 500 µl of medium. MSCBM-CD (Lonza) supplemented with 10 mmol/L dexamethasone, 10 nmol/L 3-isobutyl-1-methylxanthine, 5 mg/ml of insulin, and 60 mmol/L of indomethacin was used to induce adipogenic differentiation for 28 days, with the medium refreshed every 3 days. To evaluate the differentiation, Oil Red O solution (Sigma-Aldrich, Milan, Italy) was used to stain the lipid droplets at the cytoplasmic level. To achieve osteogenic differentiation, the hGMSCs were cultured in a 24-well plate with a density of 2 × 10^4^ cells/well with 500 µl of medium. MSCBM-CD (Lonza) supplemented with 10 nmol/L dexamethasone, 10 nmol/L beta-glycerophosphate (Sigma-Aldrich), and 50 mmol/L AA was used to induce osteogenic differentiation for 21 days. The staining that allowed us to identify the osteogenic differentiation is the Alizarin Red S (Sigma-Aldrich) solution, since it highlights the calcium deposits present in the osteogenic cells (Trubiani et al. 2016). After the staining, the differentiated hGMSCs were visualized under the Leica DM IL inverted light microscope (Leica Microsystems, Milan, Italy). The differentiation was confirmed by RT-PCR for the analysis of the expression of specific osteogenic and adipogenic genes: Runt-related transcription factor-2 (RUNX-2) and alkaline phosphatase (ALP) for osteogenesis commitment, and fatty acid binding protein 4 (FABP4) and peroxisome proliferator-activated receptor γ (PPARγ) for adipogenic differentiation. RT-PCR was performed with the TaqMan Universal PCR Master Mix according to the manufacturer’s instructions (Applied Biosystems, Foster City, CA, USA), and β-2 microglobulin (B2M Hs99999907_m1) (Applied Biosystems) was used for template normalization. To better elucidate the osteogenic differentiation, cells were processed for immunofluorescence staining as reported in the following section “Immunofluorescence analysis and confocal laser scanning microscopy”. Primary monoclonal antibody anti-human RUNX2 (1:500; Santa Cruz Biotechnology, Santa Cruz, CA, USA) was used, followed by Alexa Fluor 488 conjugated as secondary antibodies (1:200; Thermo Fisher Scientific, Life Tech., Monza, Italy), and the cells were then observed under a confocal microscope.

### hGMSC endothelial differentiation

To induce endothelial differentiation, the cell culture was stimulated with endothelial growth medium (EGM-2, Lonza) composed of basal endothelial medium-2, R3-insulin-like growth factor-1 (R3-IGF-1), growth supplements containing hydrocortisone, human fibroblast growth factor (hFGF-b), human epithelial growth factor (hEGF), GA-1000, heparin, ascorbic acid, 5% fetal bovine serum (FBS), and vascular endothelial growth factor 165 (50 ng/ml) (VEGF-165) (EGM-2 Bullet Kit; Lonza). The cells were maintained at 37 °C with 5% CO_2_ by changing the medium every 3 days. After detaching the cells from the monolayer with 0.25% trypsin–EDTA (Lonza), cells were analyzed after 10 days of stimulation for endothelial differentiation. Differentiation of hGMSCs into e-hGMSCs was observed to begin with a cell confluence of 50–60%, while hGMSCs, maintained in basal medium, were used as control cells. To evaluate the tube formation capacity, differentiated cells were plated in 12-well culture plates pretreated with 300 μl/well of Cultrex^®^ Basement Membrane Extract (Trevigen, Inc., Gaithersburg, MD, USA). The capillary-like tube structures were observed under an inverted light microscope at phase contrast after 4 h of culture (Pizzicannella et al. 2018). To evaluate the endothelial commitment, e-hGMSCs were processed for immunofluorescence staining as reported in the following section “Immunofluorescence analysis and confocal laser scanning microscopy”. Primary monoclonal antibody anti-human VEGF (1:500; Santa Cruz Biotechnology) was used, followed by Alexa Fluor 488 conjugated as secondary antibodies (1:200; Thermo Fisher Scientific), and cells were then observed under a confocal microscope.

### Study design

All the following experiments were performed in triplicate. The present work was carried out with the following groups:

- Untreated hGMSCs, used as negative control (hGMSCs)
- hGMSCs treated for 24 h with 50 μg mL^−1^ AA (hGMSCs + AA)
- hGMSCs treated for 24 h with 5 μg mL^−1^ ultrapure LPS-G from *P. gingivalis* (tlrl-ppglps, InvivoGen, San Diego, CA, USA) (hGMSCs + LPS-G)
- hGMSCs co-treated for 24 h with 50 μg mL^−1^ AA and 5 μg mL^−1^ LPS-G (hGMSCs + AA/LPS-G)
- Untreated e-hGMSCs, used as negative control (e-hGMSCs)
- e-hGMSCs treated for 24 h with 50 μg mL^−1^ AA (e-hGMSCs + AA)
- e-hGMSCs treated for 24 h with 5 μg mL^−1^ ultrapure LPS-G (InvivoGen) (e-hGMSCs + LPS-G)
- e-hGMSCs co-treated for 24 h with 50 μg mL^−1^ AA and 5 μg mL^−1^ LPS-G (e-hGMSCs + AA/LPS-G)

### Immunofluorescence analysis and confocal laser scanning microscopy (CLSM)

The sample was fixed with a 4% solution of paraformaldehyde in 0.1 M PBS (Lonza) (Sinjari et al. 2019). The cells were then permeabilized with 0.5% Triton X-100 in PBS for 10 min followed by blocking with 5% skim milk in PBS for 30 min. Subsequently, the cells were incubated for 2 h at room temperature with the following primary antibodies: anti-p300 (10 μg/mL; OriGene, Rockville, MD, USA) and anti-DNMT1 (1:100; OriGene). Then, cells were incubated for 1 h at 37 °C with Alexa Fluor 568 red fluorescence conjugated as secondary antibodies (1:200; Molecular Probes, Thermo Fisher Scientific). Subsequently, cells were stained for 1 h with Alexa Fluor 488 phalloidin green fluorescent conjugate (1:400; Molecular Probes) to mark cytoskeleton actin and for 1 h with TOPRO (1:200; Molecular Probes) to label cell nuclei. Glass coverslips were placed upside down on glass slides and mounted with ProLong Gold Antifade (Molecular Probes). At the end of the staining procedure, samples were observed using a confocal laser scanning microscope (LSM 510 Meta, Zeiss, Jena, Germany), equipped with a Plan-Neofluar oil-immersion objective (Zeiss, ×40/1.3 NA). Images were collected using an argon laser beam with excitation lines at 488 nm and a helium–neon source at 543 and 633 nm. All images were acquired at a resolution of 1024 × 1024 pixels at 12 bit (4096 grey values) using ZEN 3.0 SR software (Zeiss).

### Western blot analysis

Proteins from untreated and LPS-G- and/or AA-treated hGMSCs and e-hGMSCs were separated using sodium dodecyl-sulfate polyacrylamide gel electrophoresis (SDS-PAGE) followed by western blot analysis (Bio-Rad V3 Western Workflow™, Milan, Italy).

Membranes were saturated for 120 min at room temperature in a blocking buffer (1× TBS, 5% milk, 0.1% Tween-20) followed by overnight incubation at 4 °C with the following primary antibodies: mouse anti-p300 (1:750; OriGene) and mouse anti-DNMT1 (1:750; OriGene). Subsequently, membranes were incubated for 60 min at room temperature with peroxidase-conjugated anti-mouse secondary antibody (1:5000; Bethyl Laboratories, Montgomery, AL, USA) (Diomede et al. 2016). Enhanced chemiluminescence with the Alliance 2.7 system (Uvitec Ltd, Cambridge, UK) was used to identify and quantify the bands obtained.

### ROS labeling

hGMSCs and e-hGMSCs were seeded in a 35-mm imaging dish (µ-Dish, ibidi GmbH, Gräfelfing, Germany) and treated for 24 h in a culture medium containing 5 µg mL^−1^ LPS-G; (hGMSCs + LPS-G or e-hGMSCs + LPS-G) or 5 µg mL^−1^ LPS-G plus 50 µg/mL ascorbic acid (hGMSCs + AA/LPS-G or e-hGMSCs + AA/LPS-G) or 50 µg/mL ascorbic acid (hGMSCs + AA or e-hGMSCs + AA); or culture medium alone (hGMSCs, or e-hGMSCs). At the end of expected time, the incubation medium was removed and the cells were washed with normal external solution (NES) containing (in mM) 125 NaCl, 5 KCl, 1 MgSO4, 1 KH_2_PO_4_, 5.5 glucose, 1 CaCl_2_, 20 HEPES, pH 7.4 and incubated with 10 μM of 2′,7′-dichlorodihydrofluorescein diacetate (H2DCFDA, Thermo Fisher Scientific, Waltham MA, USA) at 37 °C in a humidified incubator (for 30 min), maintained for all procedures and respective culture media treatments. At the end of dye incubation, the cells were washed with NES and observed in NES alone (hGMSCs or e-hGMSCs), or maintained in NES plus LPS-G, LPS-G and AA, or AA alone. For each condition, confocal microscopy images were randomly acquired using a Zeiss LSM800 microscope (Zeiss), equipped with an Axio Observer D1 inverted microscope and an objective W-Plan-Apo ×40/1.3 differential interference contrast (DIC). Excitation was fixed at 488 nm, and emission collection setting of the filter was set over 505–530 nm. The acquisition settings were maintained constant between specimens.

### Methylation analysis by pyrosequencing

DNA was extracted from untreated and treated hGMSCs and e-hGMSCs by LPS and/or AA using the NucleoSpin Tissue kit (Macherey-Nagel, Düren, Germany), following the manufacturer’s instructions. DNA quantity and quality were assessed by Qubit 2.0 (Thermo Fisher Scientific).

One hundred nanograms of DNA was bisulfite-converted using the BisulFlash DNA Bisulfite Conversion Easy Kit (EpiGentek Group Inc., NY, USA). PCR amplification of chemokine (C-X-C motif) ligand 5 (*CCL5*) and intercellular adhesion molecule 1 (*ICAM1*) promoters was then performed. The PCR mix included the following reagents: KAPA HiFi Uracil Mix (Kapa Biosystems, Merck, Milan, Italy), 0.3 μM of each primer, 3 μL of converted DNA, and nuclease-free water to a final volume of 50 μL. Specific sets of primers for PCR amplification and sequencing were designed using specific software (PyroMark Assay Design version 2.0.01.15). Primer sequences (see Table 1) were designed, when possible, to hybridize with CpG-free sites to ensure methylation-independent amplification. Pyrosequencing reactions were run on a PyroMark Q96ID (Qiagen, Hilden, Germany), and CpG methylation analysis was conducted using PyroMark CpG software (Qiagen).

**Table 1:** Pyrosequencing primer sequences

Genes	Primer sequence	Sequence to analyze
CCL5-Hsa-F	5′-[Btn]AGAGTAGTGAGGGAGAGATAGA-3′	ATTAACCRATATCATAAATAAAATTACACAAAACRAAAAAAAAAACTAAAATAACCTCC
CCL5-Hsa-R	5′-[AAACATCCCCTAAAAAAAATTTCCA-3′	
CCL5-Hsa-Seq	5′-CCAAAATAACAACCAAAC-3′	
ICAM1-Hsa-F	5′-[Btn]-TTTTTTTTTTGGGAGTTGTAAAG-3′	RAACATTTCTCRAAAAACRACCAAAA
ICAM1-Hsa-R	5′-ACTAAAAAATACCCCTCCCCCTAA-3′	
ICAM1-Hsa-Seq	5′-TCCACACCTAACTAACAC-3′	

A triplicate was generated for each PCR using bisulfite-converted DNA from three different conversion reactions.

The methylation for each amplicon was calculated as the median of methylation status of each analyzed CpG. Differences in methylation pattern across samples and controls were calculated using one-way analysis of variance ANOVA, considering *p* values < 0.05 as significant.

### Statistical analysis

GraphPad Prism software (version 5.01, GraphPad Software, San Diego, CA, USA) was used for the statistical analysis. The data were analyzed using one-way ANOVA followed by Tukey’s post hoc test. *p* < 0.05 was considered statistically significant. Data were expressed as the mean ± SD. All data were collected for at least three independent experiments.

## Results

### hGMSC characterization

**Fig. 1:**
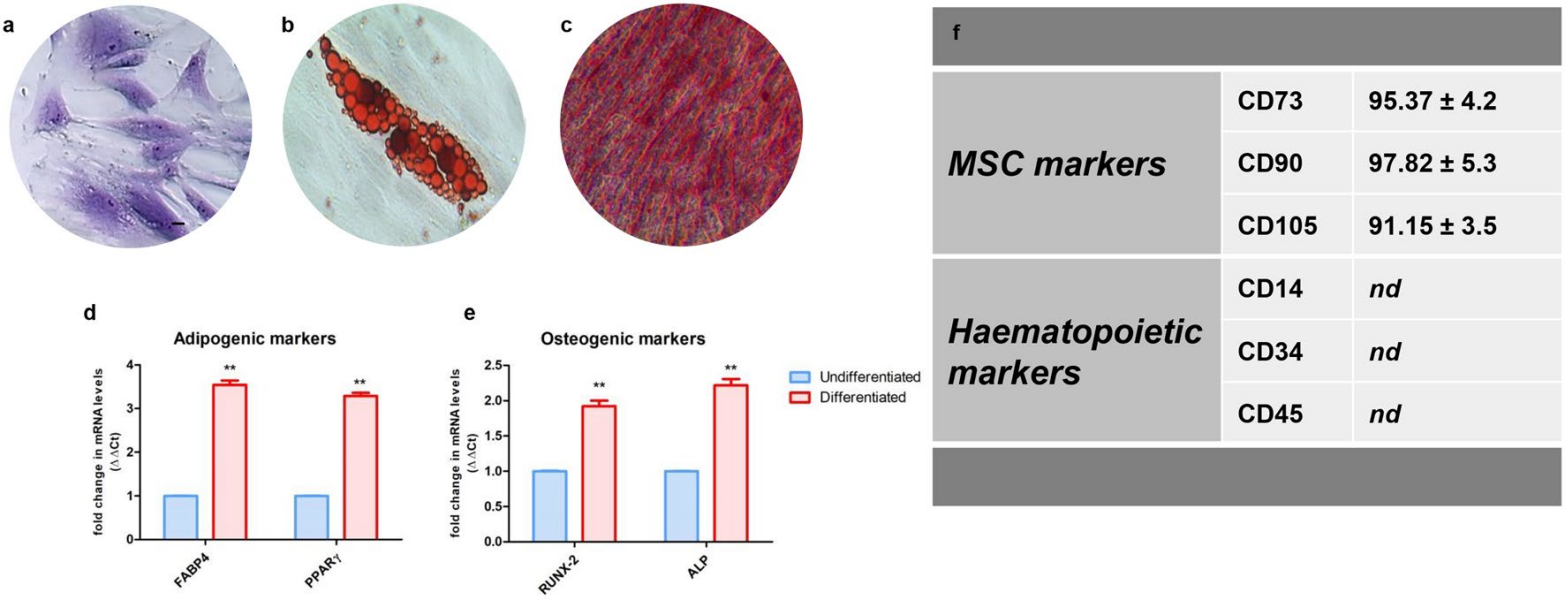
Phenotypic characterization and differentiation potential of hGMSCs. The first three images on the left show in vitro hGMSC multi-lineage differentiation: **a** morphology of the undifferentiated hGMSCs stained with toluidine blue solution and observed under a light microscope. **b** hGMSC adipogenic differentiation: red intracellular lipid droplets stained with Oil Red O solution. **c** Osteogenic differentiation of hGMSCs: alizarin red staining of calcium deposition. **d** RT-PCR graph of adipogenic markers. **e** RT-PCR graph of osteogenic markers. **f** The table summarizes the data obtained from the flow cytometric analysis. Scale bar = 20 μm. *nd* not detectable. (*N* = 5). Values are expressed as mean ± SD

hGMSCs were characterized following the established criteria of the International Society for Cellular Therapy to define their multipotent mesenchymal profile. hGMSCs able to differentiate into osteogenic and adipogenic lineages were maintained under proper culture conditions for 21 and 28 days, respectively. Cells were positive for adipo Oil Red and Alizarin Red staining, to determine the lipid vacuoles at the cytoplasmic level and calcium deposits (Fig. 1b, c). Undifferentiated cells showed a typical fibroblast-like morphology, and they are adherent on a plastic substrate (Fig. 1a).

RT-PCR data showed an increase in the mRNA expression of RUNX-2 and ALP in osteogenic committed cells, and FABP4 and PPARγ in the adipogenic differentiated samples when compared to the basal cells (Fig. 1d, e). Cytofluorimetric determination showed a positivity for CD73, 90, and 105, while cells were negative for typical hematopoietic markers, as CD14, 34, and 45 (Fig. 1f). Osteogenic differentiated cells were positive to RUNX-2 staining, the marker is localized at the nuclear level, as demonstrated by the confocal laser scanning microscopy image (Fig. S1).

### Development of vascular endothelial morphology of endothelial-committed hGMSCs

**Fig. 2:**
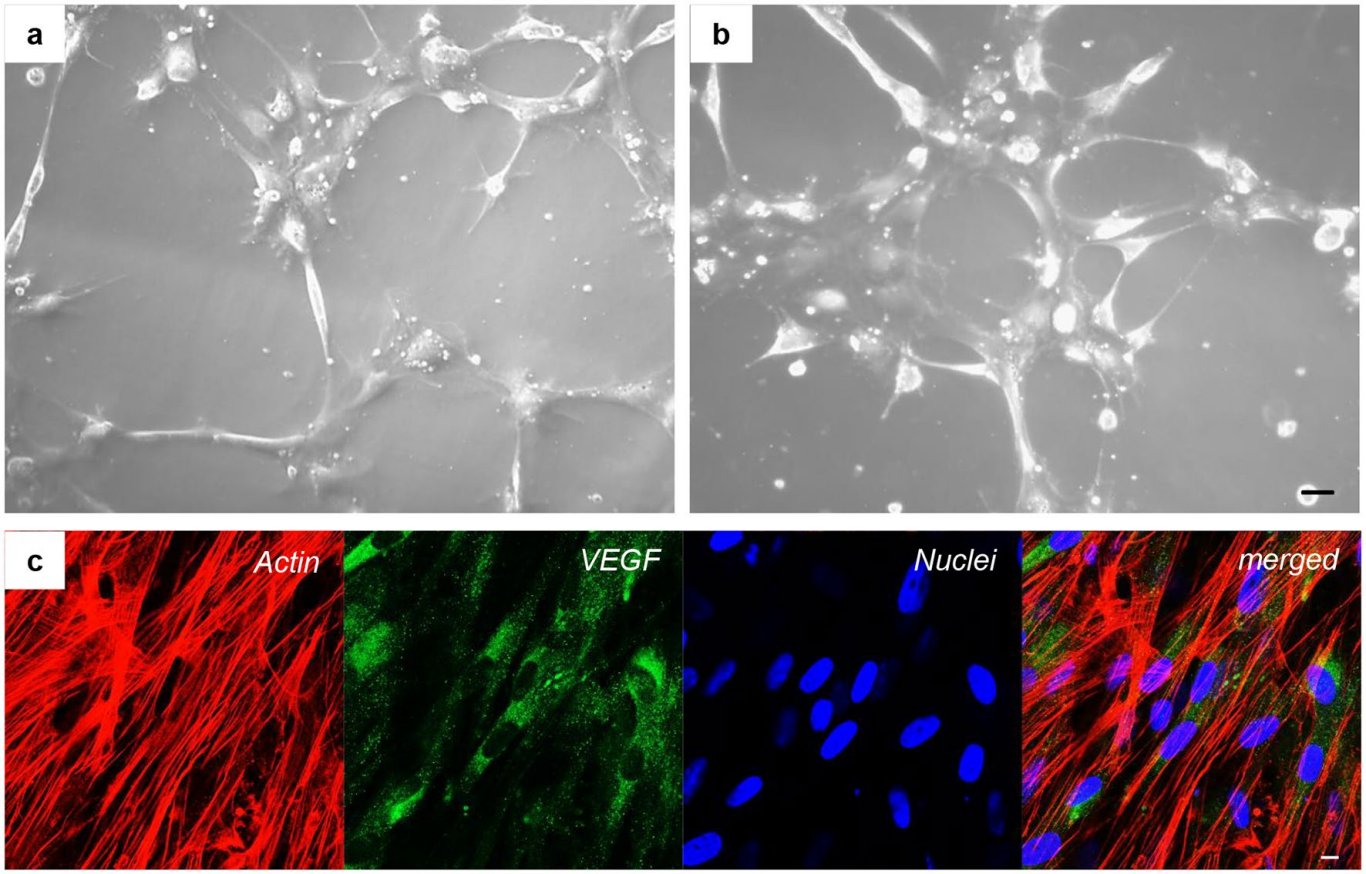
Time-lapse images of e-hGMSCs during tube formation. **a** The e-hGMSCs seeded on the Cultrex had begun to aggregate each other (1 h). **b** The cells were connected with cytoplasmic processes, developing a dense network (4 h). Bar = 20 µm. **c** Immunofluorescence staining of e-hGMSCs showed the positivity for VEGF antigen. Red: cytoskeleton actin. Blue: nuclei. Green: VEGF. Bar = 10 μm

Endothelial-committed hGMSCs were cultured on dishes covered with Cultrex gel solution. These cells were observed by light microscopy following a time course. After 1 h of culture, cells began to aggregate with each other by the establishment of connections with cytoplasmic processes (Fig. 2a), and after 4 h of culture, a mesh-like structure became a finer and denser tubular network (Fig. 2b). The evaluation of the impact of the differentiation medium kit exposure on hGMSCs to induce the endothelial transition (e-hGMSCs) was performed by examining the typical endothelial markers using VEGF immunofluorescence staining (Fig. 2c).

### LPS-G-induced modulations in p300 and DNMT1

**Fig. 3:**
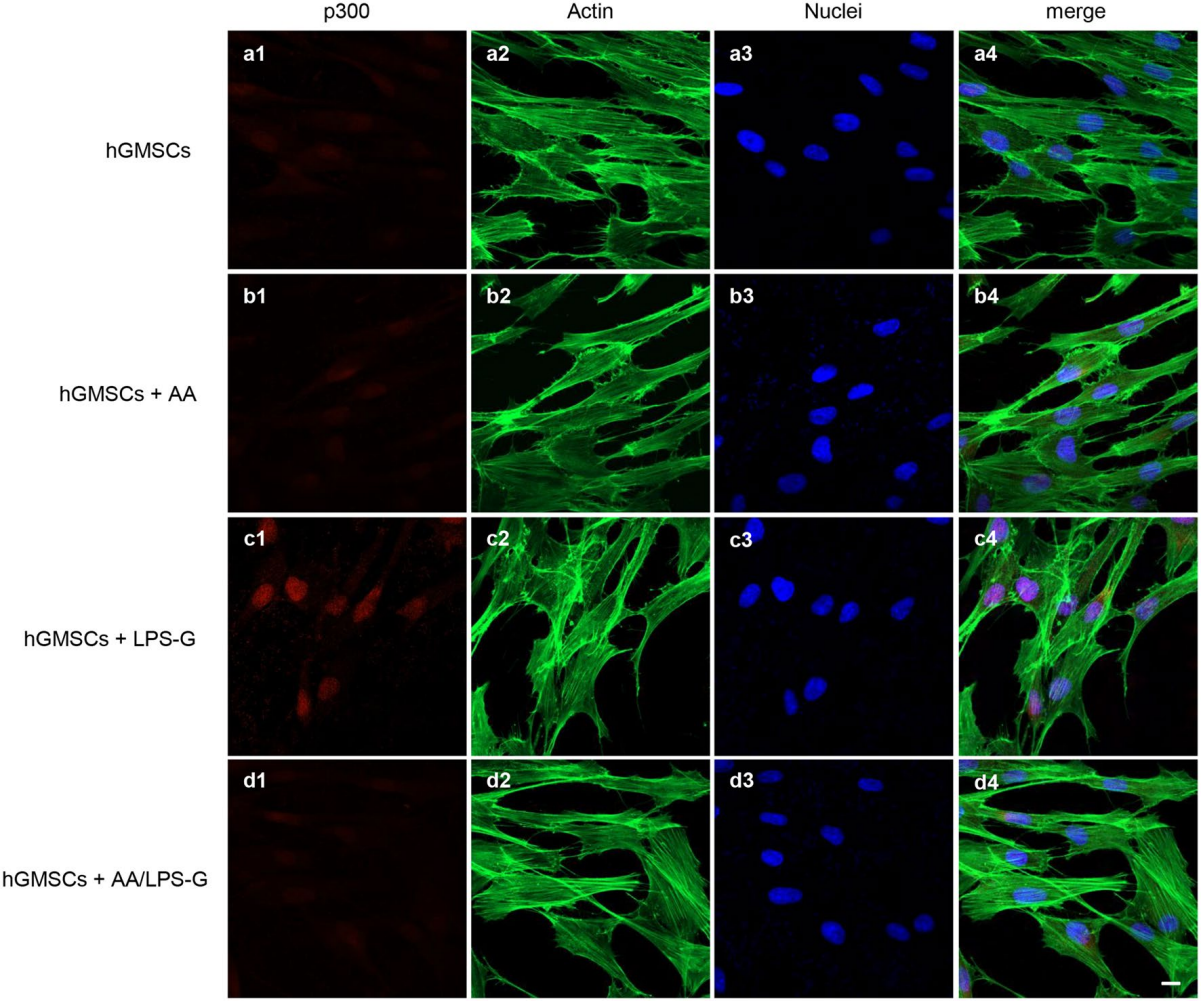
Immunofluorescence analysis of p300 expression in hGMSCs. **a1**–**a4** Untreated hGMSCs. **b1**–**b4** hGMSCs treated with AA. **c1**–**c4** hGMSCs treated with LPS-G. **d1**–**d4** hGMSCs co-treated with LPS-G and AA. Scale bar = 10 μm

**Fig. 4:**
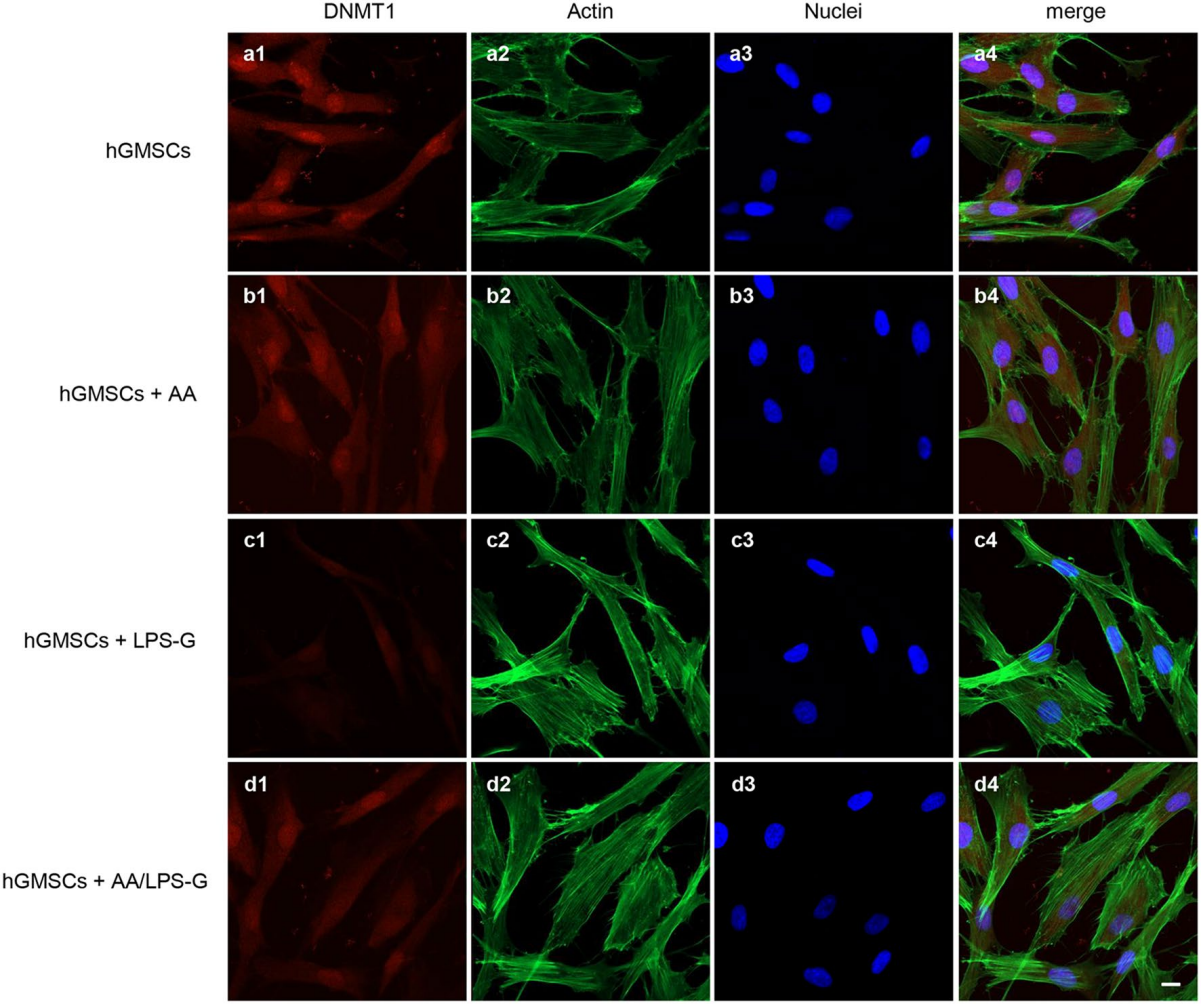
Immunofluorescence analysis of DNMT1 expression in hGMSCs. **a1**–**a4** Untreated hGMSCs. **b1**–**b4** hGMSCs treated with AA. **c1**–**c4** hGMSCs treated with LPS-G. **d1**–**d4** hGMSCs co-treated with LPS-G and AA. Scale bar = 10 μm

**Fig. 5:**
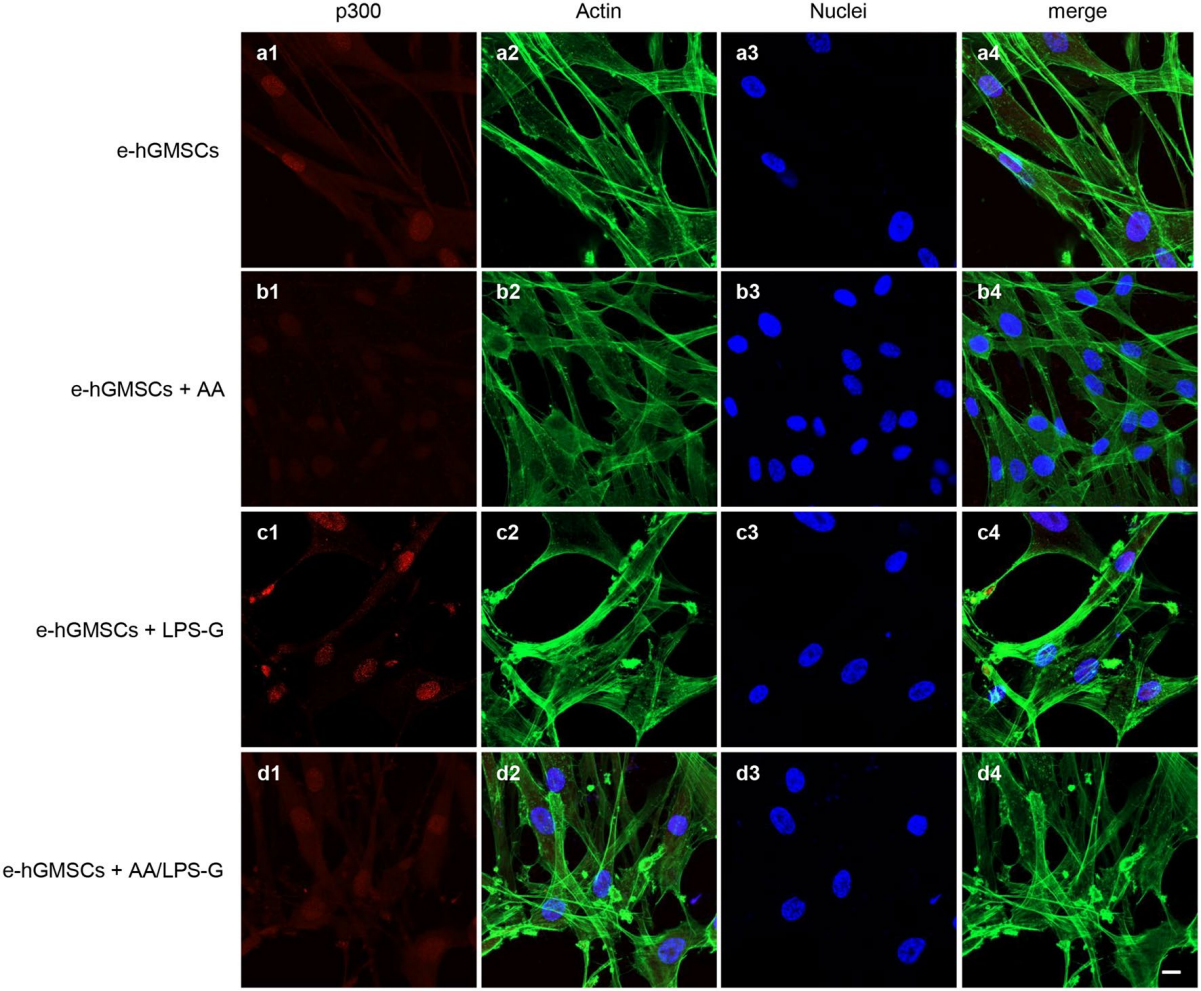
Immunofluorescence analysis of p300 expression in e-hGMSCs. **a1**–**a4** Untreated e-hGMSCs. **b1**–**b4** e-hGMSCs treated with AA. **c1**–**c4** e-hGMSCs treated with LPS-G. **d1**–**d4** e-hGMSCs co-treated with LPS-G and AA. Scale bar = 10 μm

**Fig. 6:**
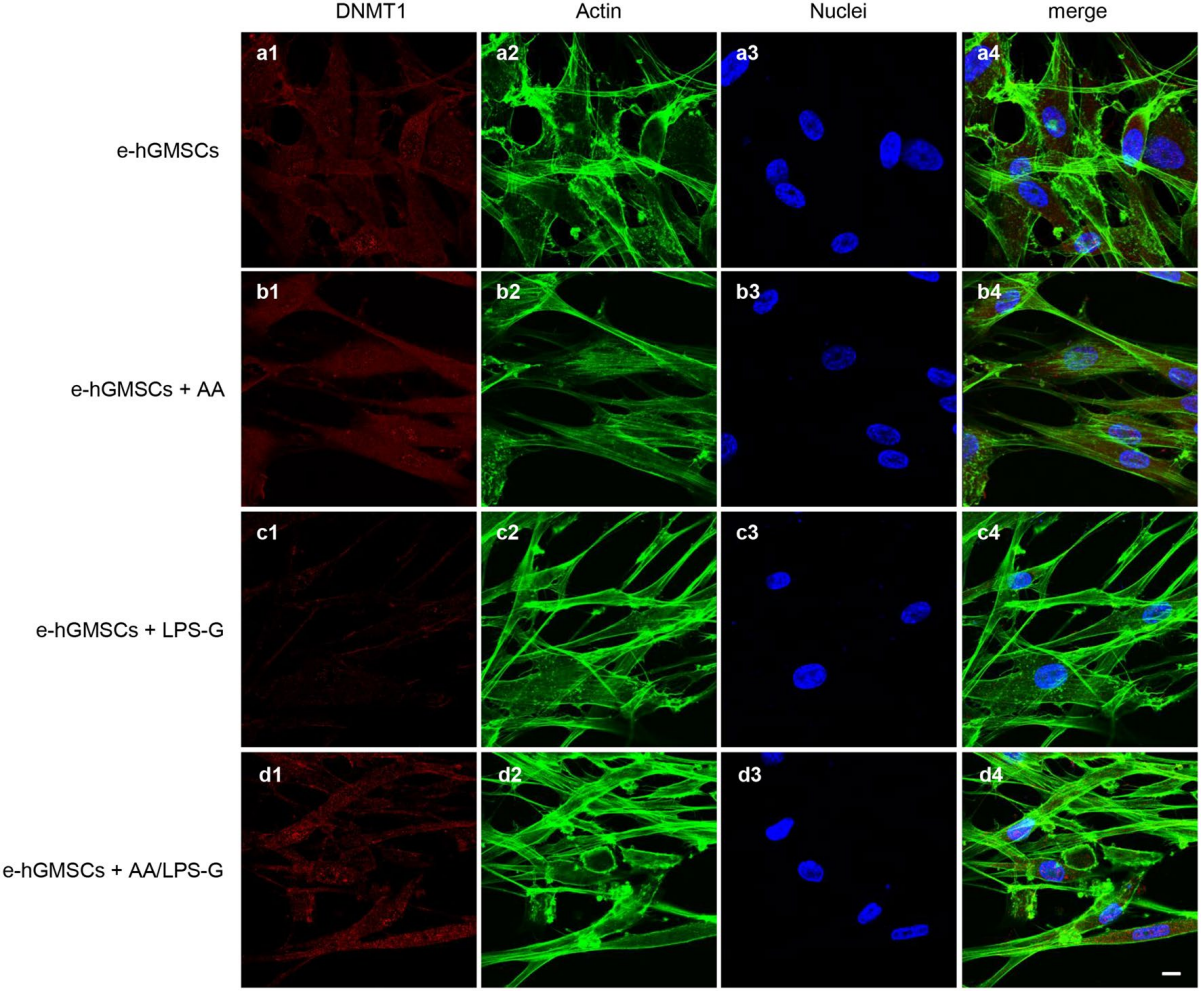
Immunofluorescence analysis of DNMT1 expression in e-hGMSCs. **a1**–**a4** Untreated e-hGMSCs. **b1**–**b4** e-hGMSCs treated with AA. **c1**–**c4** e-hGMSCs treated with LPS-G. **d1**–**d4** e-hGMSCs co-treated with LPS-G and AA. Scale bar = 10 μm

**Fig. 7:**
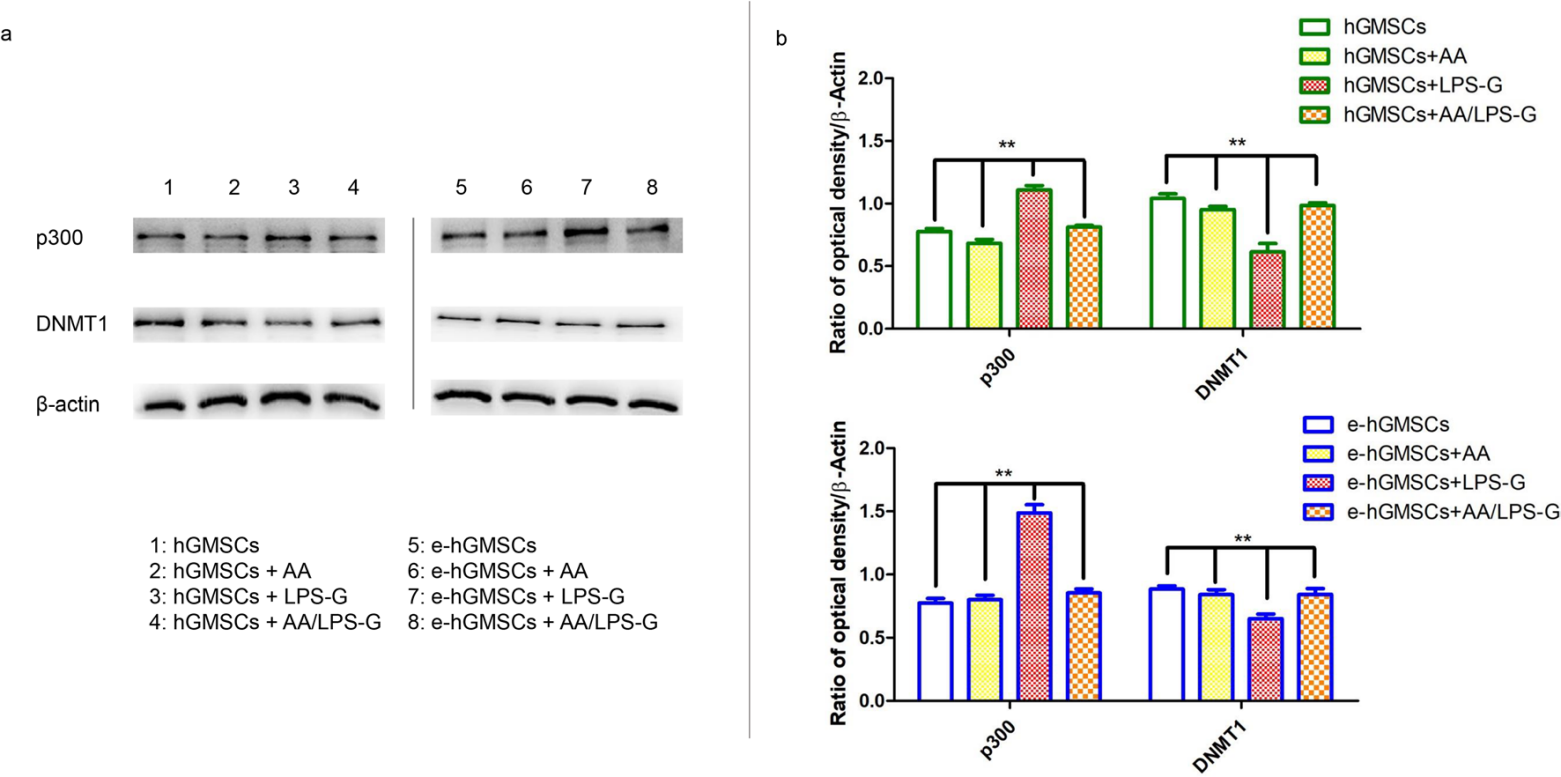
Protein levels expression. **a** Western blotting-specific bands of P300 and DNMT1 expressions in hGMSCs and e-hGMSCs treated with LPS-G alone or in co-treatment of AA. **b** Bar graphs of densitometric analysis normalized using β-actin. (*N* = 5). Values were expressed as mean ± SD

We examined whether LPS-G influenced the levels of p300 and DNMT1 in hGMSCs and e-hGMSCs. Immunofluorescence images by confocal microscopy system showed increased expression of nuclear p300 in response to LPS-G stimuli. On the other hand, DNMT1 expression was decreased in the hGMSCs and e-hGMSCs treated with LPS-G when compared to the untreated cells. The treatment with AA alone showed no differences in the expression of p300 and DNMT1 in hGMSCs and e-hGMSCs when compared to the untreated samples. Co-treatment with AA + LPS-G showed an effect in the reduction of p300 expression and an increased expression of DNMT1 when compared to the LPS-G-treated cells (Figs. 3, 4, 5, 6). These results were further confirmed by western blot analysis (Fig. 7).

### ROS localization

**Fig. 8:**
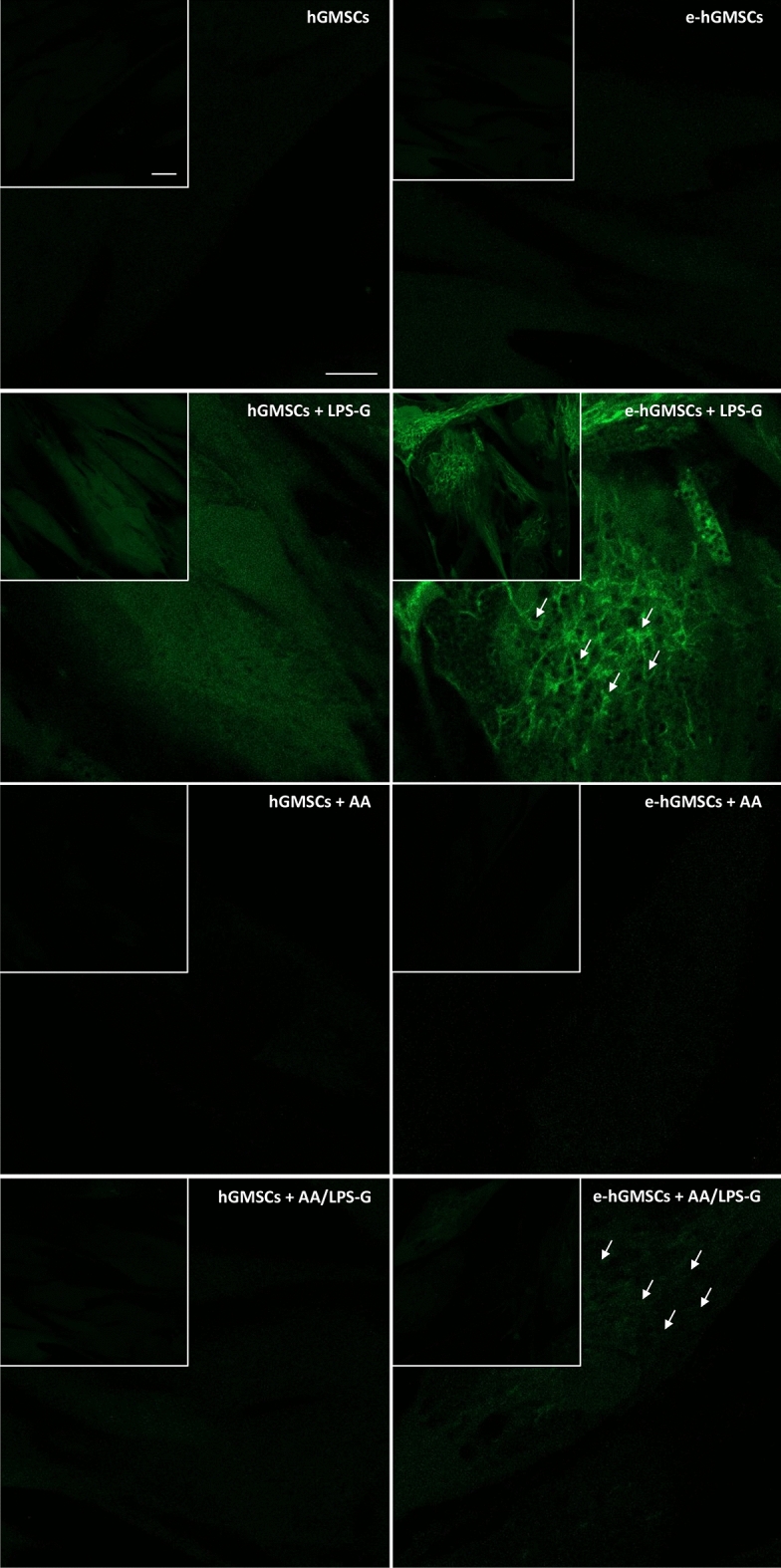
ROS measurements. Images of live cells loaded with H2DCFDA and acquired by confocal microscopy: hGMSCs, hGMSCs + LPS-G, hGMSCs + AA, hGMSCs + AA/LPS-G, e-hGMSCs, e-hGMSCs + LPS-G, e-hGMSCs + AA and e-hGMSCs + AA/LPS-G. Scale bar = 20 μm. (*N* = 5). Values were expressed as mean ± SD

ROS induced by LPS-G was visualized in hGMSCs and e-hGMSCs loaded with the cell-permeant ROS probe 2′,7′-dichlorodihydrofluorescein diacetate (H_2_DCFDA) (Fig. 8). After incubation, the probe was permeated into the cell and spread homogeneously inside the cytoplasm and organelles, where the hydrolysis of the acetate groups of H_2_DCFDA was catalyzed by intracellular esterase. This resulted in the conversion of non-fluorescent H_2_DCFDA into the fluorescence-emitting H_2_DCF, which was transformed in the highly fluorescent molecule 2′,7′-dichlorofluorescein (DCF) by ROS. Images were acquired in live cells by means of confocal microscopy, as shown in Fig. 8. In the control conditions (hGMSCs and e-hGMSCs), cells showed a low and diffused fluorescence signal, while an increase in ROS production was clearly evident in LPS-G-treated hGMSCs and e-hGMSCs. Interestingly, while in the hGMSCs + LPS-G the emitted fluorescence signal maintained a diffused pattern of emission, in the e-hGMSCs + LPS-G the increased fluorescence appeared to be more strongly localized in a “spaghetti-like” area which possibly denoted the mitochondrial structures (see arrow in Fig. 8, e-hGMSCs + LPS-G). However, addition of AA considerably reduced the LPS-G-induced ROS production in hGMSCs and e-hGMSCs.

### Methylation levels of CpGs in *CCL5* and *ICAM1*

Following statistical analysis of PyroMark Q96ID-generated data using the ANOVA test, *p*-values less than 0.05 were considered significant. No differences in methylation patterns across untreated and treated hGMSCs and e-hGMSCs by LPS-G and/or AA were observed in *CCL5* and *ICAM1* gene promoters (data not shown).

## Discussion

Oral microbiota and their products cause changes in epigenetic mechanisms and trigger inflammation, which results in the development and progression of periodontitis. When left untreated, the oral pathogens increase the proinflammatory mediators distant from their site, which leads to the development of periodontitis-associated arterial disease and CVD.

In the present study, we cultured the hGMSCs from gingival tissues of healthy patients. These cells showed expression of stem cell markers CD73, CD90, and CD105, while the cells were negative for hematopoietic markers CD14 and CD34, as previously demonstrated (Gugliandolo et al. 2018), other than the capacity to differentiate into osteogenic and adipogenic commitment as demonstrated by histochemistry staining and gene expression data. To relate the effect of LPS-G on endothelial cells, endothelial-differentiated cells derived from hGMSCs (e-hGMSCs) were studied. As previously reported, the angiogenic potential of stem cells derived from dental pulp, gingival fibroblasts, and tooth germ are able to generate endothelial tissues (Diomede et al. 2020; Pizzicannella et al. 2019). Accordingly, hGMSCs were induced to endothelial commitment, and their differentiation was evaluated by means of positivity to VEGF expression as demonstrated by immunofluorescence images and to CD31 expression, as reported previously (Pizzicannella et al. 2018). Moreover, endothelial differentiation is defined by the capacity to form a tubular structure (Barachini et al. 2014). In our study, the induced e-hGMSCs showed a network structure after seeding on Cultrex, demonstrating a capillary-like complex.

After successful establishment of hGMSCs and e-hGMSCs, the cells were exposed to LPS-G to trigger the changes in epigenetic mechanisms. Expression of histone acetyltransferase p300 and DNMT1 were investigated by immunofluorescence staining. We found that the level of p300 was increased in hGMSCs and e-hGMSCs after LPS-G treatment. Earlier studies have reported that *P. gingivalis* and *Fusobacterium nucleatum* are able to activate p300/cyclic AMP response element-binding protein (CBP) in oral epithelial cells (Liu et al. 2008). Although the precise role of p300 in periodontal disease maintenance and progression remains unclear, it is reported to regulate inflammatory NFkB signaling (Vanden Berghe et al. 1999). On the other hand, the expression of DNMT1 was decreased in LPS-G-treated hGMSCs and e-hGMSCs. Similar data were reported in a previous study where LPS exposure reduced the expression of DNMT1 in oral epithelial cells (Martins et al. 2016). LPS-G-mediated changes in the level of p300 and DNMT1 may activate the production of inflammatory mediators, which may contribute to the progression and maintenance of inflammation (Pizzicannella et al. 2021). Then, we studied the protective anti-inflammatory role of AA in LPS-G-treated hGMSCs and e-hGMSCs. AA is a powerful reducing agent and antioxidant. The efficacy of AA in reversing periodontitis and chronic gingival inflammation has been demonstrated (Chapple and Matthews 2007; Yussif et al. 2016; Diomede et al. 2019). In our study, we observed that the proinflammatory epigenetic changes caused by LPS-G were efficiently nullified by AA. Immunofluorescence staining revealed that AA markedly decreased the expression of p300 while increasing the expression of DNMT1, suggesting the anti-inflammatory role of AA. The results were further corroborated by western blot analysis. Further studies are required to better evaluate the molecular balance between DNMT1 activity and increased p300.

We then evaluated the changes in ROS levels upon LPS-G and AA treatment. Increased levels of ROS were observed in LPS-G-treated hGMSCs and e-hGMSCs, while AA reversed ROS toxicity. Our results are in line with an in vivo study where AA administration in a rat model of experimental periodontitis reduced oxidative stress (Aytekin et al. 2020), and also showed antioxidant effects in LPS-G-exposed hGMSCs and e-hGMSCs (Marconi et al. 2021).

Lastly, to support the idea that epigenetic modifications can regulate the expression of genes which influence the immune response, we analyzed the promoter methylation status of *CCL5* and *ICAM1* genes in hGMSCs and e-hGMSCs after LPS-G and AA treatment. *CCL5* is a member of the chemokine subfamily, and *ICAM1* is a member of the immunoglobulin supergene family. Both factors are known to be related to inflammatory response associated with oral pathological conditions (Sahingur and Yeudall 2015). Our data on promoter methylation analysis by pyrosequencing of *CCL5* and *ICAM1* genes did not reveal a significant modulation of CpG methylation after LPS-G and/or AA treatments in hGMSCs and e-hGMSCs. Schulz et al. studied the methylation profile of several inflammatory genes associated with periodontitis and reported that an altered methylation pattern was found only in *CCL25* and *IL17C* genes (Schulz et al. 2016). In accord with this report, our results suggest that the protective role of AA against the inflammatory process triggered by LPS-G may not be connected to modulation in DNA methylation of *CCL5* and *ICAM1* genes.

## Conclusion

Our in vitro data demonstrated that LPS-G modulated the expression of epigenetic markers p300 and DNMT1, and enhanced ROS level in hGMSCs and e-hGMSCs. AA treatment ameliorated the epigenetic changes and reduced ROS. Further studies are warranted to evaluate the molecular mechanisms underlying AA which inhibit the inflammation-related epigenetic modifications caused by oral pathogens.

## Supplementary Information

Below is the link to the electronic supplementary material.

Supplementary file1 (JPG 274 KB) Figure S1. Immunofluorescence detection of hGMSC osteogenic differentiation. Cells that underwent osteogenic commitment showed RUNX 2 positivity localized at the nuclear level. Red: cytoskeleton actin. Blue: nuclei. Green: RUNX2. Bar = 10 μm.

## Data Availability

Data are available from the corresponding author upon request.
