# Supplementary figures and images for: Role of ascorbic acid in the regulation of epigenetic processes induced by Porphyromonas gingivalis in endothelial-committed oral stem cells
Source: Histochem Cell Biol. 2021 Aug 2;156(5):423–36. doi: 10.1007/s00418-021-02014-8 (PMC8604817; doi:10.1007/s00418-021-02014-8)

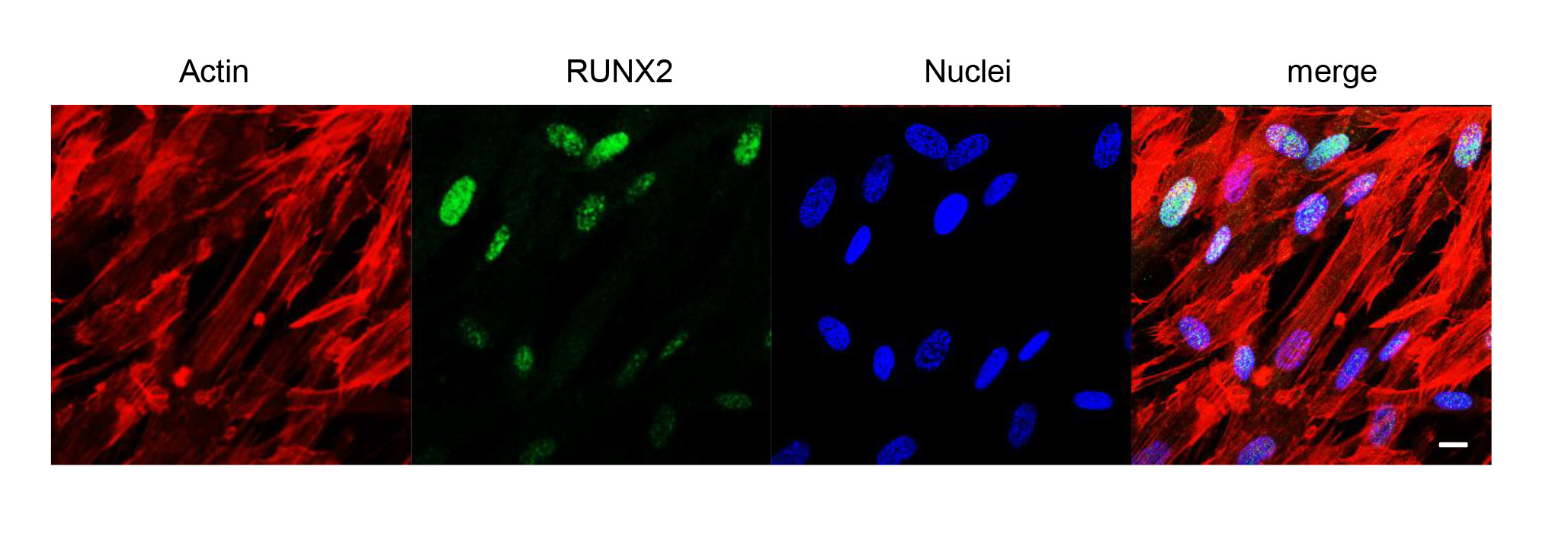

Supplement: Supplementary file 1 — Supplementary file1 (JPG 274 KB) Figure S1. Immunofluorescence detection of hGMSC osteogenic differentiation. Cells that underwent osteogenic commitment showed RUNX 2 positivity localized at the nuclear level. Red: cytoskeleton actin. Blue: nuclei. Green: RUNX2. Bar = 10 μm. [file 418_2021_2014_MOESM1_ESM.jpg]
